# Asbestos in High-Risk Communities: Public Health Implications

**DOI:** 10.3390/ijerph18041579

**Published:** 2021-02-07

**Authors:** Edward A. Emmett

**Affiliations:** Occupational and Environmental Medicine, University of Pennsylvania, Philadelphia, PA 19104, USA; emmetted@pennmedicine.upenn.edu

**Keywords:** asbestos, high-risk community, mesothelioma, vulnerable population, product ban, Four Ps of Public Health, exposure, disease registry, regulation

## Abstract

Asbestos-related diseases (ARDs)—mesothelioma, lung cancer, and asbestosis—are well known as occupational diseases. As industrial asbestos use is eliminated, ARDs within the general community from para-occupational, environmental, and natural exposures are more prominent. ARD clusters have been studied in communities including Broni, Italy; Libby, Montana; Wittenoom, Western Australia; Karain, Turkey; Ambler, Pennsylvania; and elsewhere. Community ARDs pose specific public health issues and challenges. Community exposure results in higher proportions of mesothelioma in women and a younger age distribution than occupational exposures. Exposure amount, age at exposure, fiber type, and genetic predisposition influence ARD expression; vulnerable groups include those with social and behavioral risk, exposure to extreme events, and genetic predispositions. To address community exposure, regulations should address all carcinogenic elongated mineral fibers. Banning asbestos mining, use, and importation will not reduce risks from asbestos already in place. Residents of high-risk communities are characteristically exposed through several pathways differing among communities. Administrative responsibility for controlling environmental exposures is more diffuse than for workplaces, complicated by diverse community attitudes to risk and prevention and legal complexity. The National Mesothelioma Registries help track the identification of communities at risk. High-risk communities need enhanced services for screening, diagnosis, treatment, and social and psychological support, including for retired asbestos workers. Legal settlements could help fund community programs. A focus on prevention, public health programs, particularization to specific community needs, and participation is recommended.

## 1. Introduction

As the industrial use of asbestos is phased out or eliminated, asbestos is becoming a more prominent potential cause of environmental rather than occupational disease [1,2,3]. The overall goal of this review is to address current asbestos issues with a view to the public health of communities at high risk of asbestos-related diseases (ARDs). The appreciation of public health needs should encourage research in areas that will benefit community efforts and should encourage the translation of knowledge in helpful ways. This may help avoid the “asbestos neglect” which has characterized past efforts to address the health impacts of asbestos and related elongated mineral particles (EMPs) in a broad manner [4].

This review focuses specifically on geographic communities at high risk and does not attempt to comprehensively address all potential environmental sources of asbestos to the general population. Initially, the review describes some notable and well-studied communities to illustrate the diversity of circumstances leading to the increased risk. The characteristics, challenges, and needs of these communities are then addressed from a public health perspective. Finally, a simple general framework is suggested to address and progress community public health goals.

The major ARDs are asbestosis, lung cancer, and malignant mesothelioma. Lung cancer/abnormalities may include pleural abnormalities, bronchogenic carcinomas including squamous cell carcinomas, small- and large-cell carcinoma, and adenocarcinomas [5]. In occupationally exposed cohorts, the excess of lung cancer may be greater than that of mesothelioma [6,7], but in communities ARD risk is primarily indicated by mesothelioma. Asbestos and smoking interact to cause lung cancer [8]. Malignant mesothelioma of the pleura and peritoneum is, for practical purposes, only caused by asbestos or related elongate mineral particles [9], and is unaffected by smoking status. An excess of pleural and peritoneal mesothelioma is an indisputable indicator of past asbestos exposure. However, because of their very long latency, mesotheliomas are lagging indicators of hazard. Pooling Italian and Australian data from six cohort studies of exposed workers and two of residential exposure, Reid et al. [10] found that the risk of pleural mesothelioma increased until 45 years after first exposure, when it started to plateau, with no one surviving long enough for the excess risk to disappear, while the rate of peritoneal mesothelioma continued to increase for the entire 50 years of study. The latency of lung cancer is not as long, and rates decrease 40 years after asbestos exposure ceases [7]. In contrast to the effects of occupational exposure, most community exposures appear to result in particularly high rates of mesothelioma rather than other ARDs [11].

ARDs continue to cause substantial mortality. The US CDC [12] found mesothelioma recorded on 45,221 US death certificates for ages > 25 from 1999 to 2011, with relatively small but statistically significant yearly increases over the period. The WHO estimates at least 90,000 ARD deaths annually from occupational exposure alone, with unquantifiable additional deaths from non-occupational causes [13]. Asbestos mining, importation, and use are now totally banned in over 50 countries [14], while in the United States the limited remaining uses are severely restricted, so that classical occupational asbestos exposures have declined or are declining dramatically in most developed countries. Asbestos mining and/or use does continue in Russia, China, India, and Kazakhstan [15]. While new use is increasingly absent, we are better recognizing the potential health implications of the asbestos in place from the past use of asbestos products in building construction, from the disposal or distribution of asbestos or asbestos-containing materials (ACMs), and from disturbing natural sources of asbestos.

### 1.1. High-Risk Communities

A brief review of some of the better-studied high-ARD-risk communities illustrates the variety of exposure circumstances and EMP types involved. ARD risks to communities arise from airborne asbestos fibers through a variety of pathways, including those not associated with occupation. Noonan [16] reviewed and characterized non-occupational exposure pathways into four major categories: para-occupational exposures from living in the same household as an asbestos worker from fibers bought into the residence from work [17,18], environmental exposures from industrial operations including residual neighborhood contamination, exposures to naturally occurring asbestos or related EMPs from rocks or soil, and exposures to commercial asbestos-containing products. EMPs include minerals with both an asbestiform and non-asbestiform habit [19]. Within any individual community, the environmental exposure pathways can be complex and specific to that community. A detailed characterization of pathways for Libby Montana described residential exposures from air or dust contamination in or around homes and numerous potential pathways based on lifestyle activities such as playing in or around asbestos-containing waste piles [20].

Much of our understanding of ARDs in high-risk communities derives from a small number of intensively studied locations, including Wittenoom, Western Australia; Broni, Italy; Libby, Montana; Karain, Turkey; and Ambler, Pennsylvania, whose risks resulted from a variety of industrial, community, environmental, and natural exposures [21]. 

*Wittenoom*, one of the first well-studied high-risk communities, had a poorly controlled crocidolite mining operation that closed in 1966 with substantial contamination of the nearby town [22,23,24]. High rates of ARDs were seen from occupational exposures but also from para-occupational and environmental exposures amongst town residents with no occupational exposure [25,26].

*Broni,* with approximately 10,000 inhabitants in Lombardy, was home to Italy’s second oldest and largest asbestos cement factory, which used chrysotile, crocidolite, and lesser amounts of amosite [27]. A total of 147 mesothelioma cases were diagnosed from 2000 to 2011 in Broni and surrounding towns. Using the Italian National Guidelines for attribution [28], 38 were attributed to occupational, 37 to para-occupational, and 72 to environmental exposures [27]. 

The small town of *Libby* together with neighboring Troy, Montana, was the site of the first official US Public Health Emergency declared in 2009 [29] following the discovery of very high rates of ARDs associated with mining, milling, and transporting vermiculite ore from adjacent mountains [29,30]. Both ore and product were contaminated with Libby Amphibole Asbestos (LAA), predominantly winchite, richterite, lesser amounts of tremolite, and trace amphiboles [31]. Nonoccupational LAA exposures occurred through para-occupational, residential, and lifestyle-related pathways [20]. 

Houses in the villages of *Karain* and Tuzkoy in Cappadocia, Turkey [32,33], were constructed of rock containing erionite, a natural asbestiform fiber. Mesothelioma of pleura and peritoneum were together responsible for > 50% of all deaths in these communities [34,35,36]. The extreme risk was mainly confined to members of certain families and traced to interaction between genetic predisposition and environmental erionite exposure [32,37,38]. 

*Ambler*, PA, then a small town, was once the largest asbestos-cement product manufacturing center in the US, with asbestos usage from the 1880s until 1988. First-generation immigrants and African Americans were at greatest risk of community exposures during the industrial period; there is also less information available for them, leading to an ascertainment bias as to their resultant health outcomes [39]. Presently, Ambler is a suburban area within greater Philadelphia where there are large piles of ACM waste, recently remediated and capped under the US EPA “Superfund” program. Ambler has elevated mesothelioma rates in both men and women [40]. Past lifestyle exposures associated with mesothelioma risk included playing on the asbestos poles among men and socializing with asbestos workers after work among women.

These five examples illustrate the diverse characteristics of known high-risk communities. Other communities could be added to the list. Further epidemiologic and mineralogic studies will likely expand the number of communities where local high risk is a serious concern. The use of the National Italian Mesothelioma Registry described later in this paper identified numerous additional high-risk communities, and a geospatial study of mesothelioma prevalence in Poland identified areas of mesothelioma concentration linked to asbestos cement factories [41]. Mineralogic analysis has revealed additional areas of serious EMP contamination—for example, in New Caledonia [42] and the US West [43]. Recognition that certain communities are high-risk ARD hotspots results in a need to consider implications for public health in those locations. The consideration of the diverse contributions to risk in these communities emphasizes both that there is no simple universal public health solution and also that public health responses, though they may have generalizable elements, will need to be crafted to meet the particular needs of individual communities.

### 1.2. High-Risk Communities and Public Health

Communities at high ARD risk have significant public health needs because of the serious long-term effects of asbestos (EMP) on health; community exposure; and medical, epidemiologic, administrative/regulatory, and sociologic complexities. To discuss these topics further, the major issues have been grouped in three broad categories: Firstly, distinctive features of community ARD, including demographic features, the varying expression of ARD, and vulnerable populations within a community, are topics that contribute to understanding the epidemiology of ARD within communities. Secondly, there are legal, regulatory, and policy considerations. These include acknowledging that banning asbestos mining, importation, and use will not eliminate all community risk; the shortcomings of the regulatory definition of asbestos; the diffuse administrative responsibility for community environmental exposures; the complication of diverse community attitudes to risk; and the legal responsibility for non-occupational ARD. These topics largely concern government policies, but may apply at different levels of government. Thirdly, epidemiologic and local public health programs including the use of disease registries, provision of local screening, surveillance, early diagnosis, social support services, and quantification of risks from local community exposures. These topics include the detection and provision of assistance to high-ARD-risk localities. The grouping of topics into these three areas was largely a matter of convenience; other groupings would make sense and there is some overlap between the topics. 

## 2. Unique and Distinct Features of Community Asbestos Exposue

### 2.1. Age and Gender Distribution of Community Mesothelioma

Non-occupational mesothelioma from community exposure is characterized by a higher proportion of females and a tendency towards a younger age distribution than in occupational exposure [25,27,44,45] The younger age distribution appears due to the younger age at first exposure, so at any given age there is greater opportunity to have accrued the characteristically long latent period, an effect which is greatest where exposure started in very early childhood [46]. Apart from this exposure cohort effect, children have no difference in susceptibility to mesothelioma compared with adults after adjustment for exposure and sex [47]. The observed latency for pleural mesothelioma can be longer in women than in men. Soeberg et al. [48] found that the proportion of pleural mesothelioma in Australian women slowly increased over the 1982–2009 period, whereas very high asbestos consumption peaked in 1970–1979 and declined rapidly thereafter. They attributed these findings to a longer latent period from community para-occupational and environmental exposures, reflecting the general rule that lower exposure levels are associated with longer latent periods. Reid et al. [10] observed a similar dose effect in a pooled analysis of eight cohort studies. Longer latency has also been observed in Turkish emigrants to Sweden compared with non-emigrants [36] and in Wittenoom women who worked for the asbestos company compared with those with only residential exposure [45].

### 2.2. Varying Expressions of ARD in Different Communities

The pattern of ARD in any particular community is influenced by amount of exposure, age at exposure, fiber type, and genetic predisposition. The highest, predominantly occupational, exposures are associated with excess lung cancer and asbestosis and a higher proportion of peritoneal mesothelioma than pleural mesothelioma. At lower non-occupational exposures, pleural mesothelioma predominates [10].

ARDs may have other distinctive features in different communities, with fiber type seemingly being a major factor. A follow-up study of crocidolite-exposed Wittenoom children (aged < 15 years) found excess ovarian and brain cancers in women and leukemia, prostate, brain, and colorectal cancers and all-cause mortality in men, in addition to excess mesothelioma [46]. ARDs from LAA seem to have distinctive features. Occupational exposure to vermiculite and/or residence in Libby is associated with mesothelioma [49,50] and ARD mortality [51], but also appears to provoke more progressive pleural disease than other fiber types [52]. Other unusual features include increased frequency of antinuclear autoantibodies (ANAs) [53,54] and systemic autoimmune disease [55], which have not described in other asbestos-exposed groups. Experimentally, LAA induces mesothelial cell autoantibodies (MCAAs), collagen deposition both in vitro [56] and in vivo [57], and laminar pleural radiographic changes [58]. In contrast, chrysotile does not induce autoantibodies in mice or humans, while MCAAs are less frequent and are not associated with pleural disease [54,59].

### 2.3. Vulnerable Groups within Communities

Within communities, social, behavioral, extreme event, or genetic predisposition vulnerabilities for asbestos exposure and/or ARD may deserve focussed public health interventions; the need varies from community to community.

*Social vulnerability* can occur in those who are unwittingly exposed to asbestos or are ignorant of the associated risk. In many developed countries, there is generally high awareness of the hazards from asbestos, particularly in the skilled trades and amongst unionized employees; however, there is much less awareness of the presence and potential hazards from community exposure sources. This deficit will need to be addressed by educational and promotional campaigns to raise awareness and understanding of EMP-related risks across communities. Particular subgroups within the community may be more at risk, such as those who do not speak the dominant language and ethnic, tribal, and environmental justice groups. Overall, there is a paucity of literature about the socioeconomic determinants of ARD within communities to guide these efforts.

*Behavioral vulnerabilities* arise from activities that increase the probability of encountering particular pathways of asbestos exposure or which increase ARD risks given the same exposure. Children and adolescents may experience distinctive high exposure risks. In Ambler, adolescents had a distinct set of exposures to ACM from playing in waste sites and recreational activities in abandoned factory sites, where they dismantled fencing and ignored signage to access ACM waste areas [39]. Children in Libby Montana had substantial LAA exposures during outdoor activities and reported playing in vermiculate waste piles “all the time” [60]. Adult behaviors can increase risk; Olsen et al. [61] described significantly increased mesothelioma in Australian do-it-yourself home renovators, presumably related to exposure to ACM in residential reconstruction. Smokers have additional vulnerability to lung cancer from the multiplicative effects of smoking and asbestos.

*Impacts from Extreme Events* have been described. The terrorist destruction of the World Trade Center led to the dissemination of asbestos fibers locally and consequent exposures to first responders and residents [62]. Weather events such as flooding distributed asbestos fibers from ACM waste sites, into residential areas in Ambler [39].

*Genetic vulnerability* to mesothelioma may be extreme, as in Karain [37]. Familial clustering in other settings raises the question of whether it is a consequence of shared para-occupational exposures or shared genetic vulnerability. In Wittenoom, De Klerk et al. [63] used a survival model to estimate the expected number of mesotheliomas in family groupings. The risk ratio for blood relatives compared with non-blood relatives was 1.9 (95% CI 1.3 to 2.9, *p* = 0.002), suggesting an important though not dominant genetic component, and much lower than the estimates of Roushdy-Hammady et al. [37] for Turkish village erionite exposures.

Community pathways provide asbestos exposure to groups of people who are not usually in the workforce, such as pregnant women, the seriously ill, and the aged. To date, there is no convincing evidence that these groups, nor any ethnic group in the general population, has an increased predisposition to ARD given equivalent exposures, but the available data to resolve these questions are sparse.

## 3. Legal, Regulatory and Policy Considerations

### 3.1. Banning Asbestos Mining, Import and Use Does Not Eliminate Community Risk

To prevent ARDs, we must prevent asbestos exposure. The International Labor Office (ILO) has called for national bans on asbestos: it’s 95th Resolution of June 1, 2006, promotes the elimination of the future use of all forms of asbestos and asbestos-containing materials in all member states [64]. There is an undeniable and urgent need for such bans, which many countries have already implemented [14]. However, although banning the import, export, and use of asbestos will effectively prevent ARD from being mining and the manufacturing of new asbestos-containing products, it will not, in and of itself, eliminate hazards from asbestos in place in contaminated communities or from natural asbestos fiber sources. The effective prevention of hazards from asbestos in place will still be needed in addition to measures to reduce its future use or sale. 

### 3.2. Shortcomings of the Regulatory Definition for Asbestos

Asbestos is a generic term describing a number of elongated silicate minerals that produce thin, flexible fibers when crushed and which have a high tensile strength and resistance to heat and chemicals [65]. Six types of asbestos are currently strongly regulated; those which were in commercial use in 1975—namely, the serpentine mineral chrysotile and five amphibole minerals: riebeckite asbestos (crocidolite), cummingtonite grunerite asbestos (amosite), tremolite asbestos, actinolite asbestos, and asbestiform anthophyllite [66]. For the purposes of the US EPA, US Occupational Safety and Health Administration (OSHA), and the WHO, asbestos minerals are regulated and controlled if they are longer than 5 µm and have a length to width (aspect ratio) of at least 3:1 [65]. There is no strict minerologic definition to correspond with this regulatory definition [67] and the current definition of asbestos is quite inadequate [19,68,69]. This definition may have been appropriate for regulations controlling occupational workplace exposures to asbestos from industrial operations, but is inappropriate for many high-risk community exposures. The communities of Karain and Libby exemplify human risk from erionite (fibrous zeolite), and LAA (predominantly winchite and richterite) respectively. Erionite is strongly carcinogenic in laboratory animals [32,70,71] and has been assessed by IARC as a Group1 human carcinogen [72]. Similarly, fluoro-edenite [73] another asbestiform amphibole is responsible for excess mesothelioma in Biancavilla, Italy [74,75]. Non-regulated carcinogenic asbestiform fibers such as erionite have wide natural distribution across the US West [76] and could readily be disturbed in a variety of ways. Amadeus et al. [77] and Sullivan [78] have described how LAA in vermiculite from Libby was transported, processed and used across the US, Canada and elsewhere including as in loose-fill insulation and agricultural products. From the point of view of community hazard, regulation of both occupational and environmental health exposures should encompass all fibers capable of producing mesothelioma and other ARD. Revising the definition of EMP/asbestos for regulatory applications will involve toxicologic, mineralogic and analytic considerations, likely a protracted process. If this task proves insurmountable, regulatory coverage should at the least be extended to include erionite, fibrous-edenite, winchite and richterite.

### 3.3. Diffuse Administrative Responsibility for Control of Community Environmental Exposures

When asbestos is primarily an occupational hazard, the lines of administrative responsibility to protect workers’ health are usually clear: there is a single responsible party, the employer, and a single enforcing agency, in the US the Occupational Safety and Health Administration (OSHA). The general duty clause of the US Occupational Safety and Health Act states that “each employer (1) shall furnish to each of his employees a place of employment which is free from recognized hazards that are causing or are likely to cause death or serious physical harm to his employees and (2) shall comply with occupational safety and health standards promulgated under this Act”. The standard for asbestos has detailed requirements for permissible exposures, risk identification and assessment, engineering controls, work practices and respiratory protection, proper hazard communication, demarcation of areas where there are risks, separate decontamination and luncheon areas, training requirements, medical surveillance, and record keeping 1994 [66]. Most other countries have similar occupational health and safety regulations covering asbestos. Compliance with these regulatory and enforcement regimes should also serve to prevent para-occupational exposure through decontamination requirements, such as showering and changes of clothes after work shifts.

In contrast, for many potential environmental and lifestyle-related exposures the chain of responsibility is very complex. Emmett and Cakouros [21] describe responsibilities for the Bo-Rit Asbestos Superfund site near Ambler, an area of approximately 25 acres including contaminated creeks and parkland. The complete removal of ACM was considered impractical in part because of the large volume, so remediation occurred through the capping of the waste with clean materials and with associated administrative controls such as restrictions on land use [79]. The US EPA was responsible for remediation and hazard removal on the superfund site but did not have the automatic right of access to surrounding private properties where additional contamination was possible. After remediation, EPA monitors long-term effectiveness but relinquishes site responsibility to the Pennsylvania Department of Environmental Protection. Subject to some restrictions, future use, control of access, and measures to prevent or minimize asbestos exposure will be variously controlled by three different current landowners, the Pennsylvania Department of Environmental Protection, three different local municipalities, and a County Planning Department. Other important stakeholders with various roles include neighborhood residents and businesses, the State Health Department, and a federal health agency (Agency for Toxic Substances and Disease Control). In other community sites, the parties involved may vary but responsibility is often equally diffuse. This situation is quite different to that in industry where there is usually a single line of authority. Sustainable control of long-term environmental exposure risks where many parties share limited responsibilities requires consistent and coordinated activities and a shared common goal. If there are divergent views amongst major stakeholders, achieving appropriate hazard control over the long term will likely be contentious, prolonged, and ineffective. 

### 3.4. Diverse Community Attitudes to Risk and Prevention

How divergent are views within high-risk communities about environmental asbestos hazards? In depth interviews of stakeholders around Ambler Superfund Asbestos sites revealed very wide-ranging views about risk and appropriate future uses for the area, these community views also changed over time [80]. Human behavior favors ignoring or suppressing concerns about risk [81]: raising questions of how best to effectively “memorialize” future environmental risk from areas such as capped ACM waste sites, how to effectively counter lifestyle activities which increase exposure to ACM, and how to discourage illegal asbestos dumping [82]. More research is needed to understand the genesis of diverse attitudes to risk and to effectively communication risk to communities to enhance problem solving and the achievement of a consensus around acceptable, sustainable risk reduction. Diverse views and diffuse responsibilities highlight the importance of social marketing to generate constructive community prevention. The thrust of social marketing is to apply modern marketing to the preventive health activities, particularly at the community level [83]. In this approach, the four Ps of marketing (product, place, price, promotion) are replaced by prevention, programs, particularization, and participation [84]. When applied to asbestos/EMP, prevention entails controlling and eliminating future asbestos exposure through all applicable pathways. Programs are delivered to educate the community; identify particularly vulnerable groups; and provide access to screening, treatment, and social groups. Particularization refers to tailoring programs to the needs, specific diseases, exposure characteristics, and culture of the community. Participation refers to ensuring diverse input, ownership, and involvement across the community. 

### 3.5. Legal Responsibility and Compensability of Non-Occupational ARD

Legal recourse for compensation for occupational ARD has been relatively straightforward in many jurisdictions; successful litigation has led to the establishment of large trusts, funded by industrial users or suppliers of asbestos. These funds may also compensate non-occupational ARD where responsibility for the causal exposure can be established. However, establishing a single responsible party may be difficult. Ndlovu [85] found that most South Africans with community-derived mesothelioma had not received compensation despite the existence of numerous asbestos trust funds. Marinaccio et al. [86] reported concern about Italian insurance and welfare protection for those with community exposure caused mesothelioma, noting that different pathways of non-occupational exposure posed different issues with respect to acceptability under the welfare protection framework. Gordon and Leigh [87], citing ARD risks to non-professionals exposed to asbestos cement products in home renovation or other do-it-yourself activities, maintained that manufacturers of asbestos-containing products have a continuing duty of care to inform users about asbestos risks. Controversial legal issues include a claimant’s ability to prove that the manufacturer could and should have taken steps (before the time of exposure) to have drawn the risk to the user’s attention. It may be difficult to prove that a particular exposure caused or contributed to cancer manifesting many years later. The multiple and diffuse responsibilities for control of community and non-occupational environmental and natural fiber exposure referred to above will likely challenge attribution of liability. Legal settlements might usefully incorporate funding of future efforts to eliminate further community exposure and to fund the community support programs addressed later in this paper.

## 4. Epidemiologic and Local Public Health Issues

### 4.1. Use of Disease Registries

National Mesothelioma Registries help to identify communities at high risk and may overcome common deficits in surveillance, including underreporting, lack of standardized diagnostic criteria, poor elucidation of occupational and environmental exposures, and less than comprehensive coverage [88]. According to van Gerwen et al. [89], five countries currently operate a mesothelioma-specific registry, including Italy, France, the UK, Australia. and South Korea, all but the UK use interviews to collect exposure data. Registries have made many contributions to our knowledge of ARD in communities. The Italian National Register of Malignant Mesothelioma (ReNaM) has recorded mesotheliomas and collected asbestos exposure information from 1993 to the present for most of Italy [44,86]. A national study using ReNaM was able to identify distinct geographic clusters in communities where asbestos-cement plants had operated (Broni discussed above, Casale Monferrato, and Bari), and sites of shipbuilding and repair (Monfalcone, Trieste, La Spezia, Genova, Castellamare di Stabia, Livorno, and Taranto). A cluster was also identified in Biancavilla, Sicily, where naturally occurring asbestiform fluoro-edenite was used in construction and road paving [86]. Even in prior sites of asbestos-cement plants, where the industrial use of asbestos had been broadly similar, the relative proportions of mesotheliomas attributed to all nonoccupational exposures and to para-occupational, residential, and leisure-related environmental pathways, respectively, varied from town to town. A French National Registry has operated since 1998 [90]; the use of a panel of pathologists to achieve diagnostic consensus showed that only about 2/3 of submitted diagnoses could be confirmed, indicating the importance of strict criteria and data review [91]. Australian mesothelioma data have been collected under two successive registry schemes [92]: the Australian Mesothelioma Surveillance Program (1979–1985) and the Australian Mesothelioma Register (1986–2002 and 2010-present). Additional exposure data in the Australian Registry enabled the recognition of new at-risk groups, which was facilitated by a voluntary asbestos exposure questionnaire with residential and occupational history and information about exposures outside paid work [93], resulting in the recognition of high mesothelioma incidence for do-it-yourself home renovators. Mesothelioma registries incorporating standardized diagnostic criteria and exposure data enable monitoring of trends in incidence attributable to different exposures in different regions of a country and should be increasingly important as the relative proportion of mesothelioma from community exposure increases.

### 4.2. Local Screening, Surveillance, Early Diagnosis and Social Support Services

High-risk communities may need enhanced services to provide screening, prompt diagnosis, and treatment for ARD and social and psychological support. These services may also be necessary for retired occupationally exposed workers as well as for the general community. In some jurisdictions, EMP-exposed workers may lose access to employer sponsored surveillance and other programs upon retirement. Yet, because of the long latency for lung cancer and mesothelioma, the greatest risk of disease may occur after work with asbestos has ceased. This is the situation in the US, where Occupational Health and Safety regulations require employers to provide periodic medical evaluations for those working with asbestos, but require no follow-up or surveillance once the worker leaves the job. The burden of any help or surveillance for these individuals thus becomes the responsibility of public health authorities.

Effective screening could facilitate early diagnosis, leading to improved treatment and enhanced cure/survival rates for lung cancer and mesothelioma. With a major focus on lung cancer, Khattab et al. [94] developed a useful tool to predict which former asbestos workers would benefit most from prospective surveillance. The prediction incorporated the risk factors of age, exposure duration, time since first exposure, age at first exposure, job (partly as a proxy for degree of exposure). and smoking. Screening for mesothelioma poses an additional challenge: the current lack of demonstrable benefit from early detection. The prognosis for mesothelioma remains poor with mean life expectancy still measured in months. Using US National Surveillance, Epidemiology, and End-Results (SEER) data from 1973 to 2009, Taioli et al. [95] found no significant improvement in mean survival over the past four decades. More optimistic scenarios may be emerging, as improvements of a few months increased survival for pleural mesothelioma have been reported from the Netherlands [96] and Australia [48] corresponding with introduction of new chemotherapeutic regimes. The lack of personal benefits from screening affected the enthusiasm of Karain villagers: approximately 50% participated in free screening, many of those who did not participate believed screening would be useless unless there was effective treatment to offer [32]. Improvements in treatment, imaging, or biomarker developments [97,98] might allow detection at an earlier, more treatable stage. In this eventuality, a model for mesothelioma risk might help identify both communities and individuals within a community by identifying who would have the greatest benefit from screening and early intervention.

A multi-faceted model community program has operated in Libby Montana since 2000, following the discovery of increased mortality [30]. Managed through a local Board of Directors, the Libby Center for Asbestos Related Diseases (CARD) provides community health screening, patient care, social service support, and counseling to individuals and families. Since 2009, electronic medical records have been used. These activities have engendered remarkable community acceptance and local support. The population followed regularly now exceeds 5000. The evaluation of the utility of programs of this type will provide guidance for providing services to other high-risk communities.

### 4.3. Quantifying Risks from Community Exposure

Quantifying the risk of non-occupational community exposure pathways is challenging for several reasons. Data on historic levels of exposure are generally sparse or absent for community exposure pathways. Reconstructing exposures from measurements made in the distant past can be highly inaccurate because of serious historic methodologic limitations to measurement, as described by Rogers [99]. Multiple potential exposure pathways were common: occupational exposures was usually accompanied by additional environmental exposure. Reliance on medical histories or questionnaires to identify past asbestos exposure is imperfect: Leigh and Driscoll [92] found that 19% of subjects with mesothelioma in the Australian Mesothelioma Registry gave no history of asbestos exposure, but when lung tissue was examined 81% of this group had fiber counts >200,000 fibers/g dry lung. This finding suggested that they were asbestos-exposed but could not recall or had never recognized the exposure circumstances. Accordingly, it is usually difficult or impossible to develop a dose–response relationships for single non-occupational exposure pathways in isolation. The extrapolation of exposure results from one community to another is also challenging because differences in EMP type, climate, rainfall, local habits, and cultures may greatly modify the cumulative “dose” received from non-occupational exposure pathways. Because of these multiple uncertainties in characterizing risk from past and present community exposures, we will need to be prudent in risk assessment to include all potential sources of risk when remediating or removing community asbestos sources.

## 5. Conclusions

A number of sentinel communities have been identified worldwide with high risks of ARD, particularly mesothelioma; more are emerging as more attention is given to community rather than occupational exposures. These communities represent a significant and expanding public health concern whose resolution requires effective action at both the national/international policy level and the local community program level.

At the broad policy level, a regulatory regime that defines asbestos/EMP in a manner that comprehensively covers the carcinogenic and ARD risk; recognition that banning the mining, importation. and use of asbestos will not eliminate all risk in communities; steps to improve the coordination of the many agencies concerned with the prevention of environmental asbestos/EMP exposure; and specific registries to identify communities at increased risk of ARD, particularly mesothelioma, are indicated.

At the community level, education and social marketing to enhance awareness and understanding of ARD risk and prevention; attention to the special needs of vulnerable groups; programs for screening, surveillance, early diagnosis, and social support; as well as an appreciation that the long-term risks are difficult to quantify are required. Because of the large variability in many dimensions between affected communities, these activities will need to be crafted to the needs and circumstances of individual communities. A Four Ps Public Health Focus is suggested to help these communities, with an emphasis on prevention, programs, particularization, and participation. Where feasible, legal settlements might usefully deliver funding to provide the required community programs.

## Data Availability

Not Applicable.
